# TAK-994 mechanistic investigation into drug-induced liver injury

**DOI:** 10.1093/toxsci/kfaf003

**Published:** 2025-01-09

**Authors:** Tadahiro Shinozawa, Kazumasa Miyamoto, Kevin S Baker, Samantha C Faber, Ramon Flores, Jack Uetrecht, Christian von Hehn, Tomoya Yukawa, Kimio Tohyama, Harisha Kadali, Marcin von Grotthuss, Yusuke Sudo, Erin N Smith, Dorothée Diogo, Andy Z X Zhu, Yvonne Dragan, Gvido Cebers, Matthew P Wagoner

**Affiliations:** Takeda Pharmaceutical Company Ltd, Fujisawa, Kanagawa 251-8555, Japan; Takeda Pharmaceutical Company Ltd, Fujisawa, Kanagawa 251-8555, Japan; Takeda Development Center Americas, Inc, Cambridge, MA 02139, United States; Takeda Development Center Americas, Inc, San Diego, CA 92121, United States; Clarivate, 398 08025 Barcelona, Spain; Department of Pharmacology and Toxicology, Temerty Faculty of Medicine, University of Toronto, Toronto, ON M5S 3M2, Canada; Takeda Development Center Americas, Inc, Cambridge, MA 02139, United States; Takeda Pharmaceutical Company Ltd, Fujisawa, Kanagawa 251-8555, Japan; Takeda Pharmaceutical Company Ltd, Fujisawa, Kanagawa 251-8555, Japan; Takeda Development Center Americas, Inc, Cambridge, MA 02139, United States; Takeda Development Center Americas, Inc, Cambridge, MA 02139, United States; Takeda Pharmaceutical Company Ltd, Fujisawa, Kanagawa 251-8555, Japan; Takeda Development Center Americas, Inc, San Diego, CA 92121, United States; Takeda Development Center Americas, Inc, Cambridge, MA 02139, United States; Takeda Development Center Americas, Inc, Cambridge, MA 02139, United States; Takeda Development Center Americas, Inc, Cambridge, MA 02139, United States; Takeda Development Center Americas, Inc, Cambridge, MA 02139, United States; Takeda Development Center Americas, Inc, Cambridge, MA 02139, United States

**Keywords:** drug-induced liver injury, hepatotoxicity, drug discovery, covalent binding, bile salt efflux pump

## Abstract

The frequency of drug-induced liver injury (DILI) in clinical trials remains a challenge for drug developers despite advances in human hepatotoxicity models and improvements in reducing liver-related attrition in preclinical species. TAK-994, an oral orexin receptor 2 agonist, was withdrawn from phase II clinical trials due to the appearance of severe DILI. Here, we investigate the likely mechanism of TAK-994 DILI in hepatic cell culture systems examined cytotoxicity, mitochondrial toxicity, impact on drug transporter proteins, and covalent binding. Hepatic liabilities were absent in rat and nonhuman primate safety studies, however, murine studies initiated during clinical trials revealed hepatic single-cell necrosis following cytochrome P450 induction at clinically relevant doses. Hepatic cell culture experiments uncovered wide margins to known mechanisms of intrinsic DILI, including cytotoxicity (>100× *C*_max_/IC_50_), mitochondrial toxicity (>100× *C*_max_/IC_50_), and bile salt efflux pump inhibition (>20× *C*_ss, avg_/IC_50_). A potential covalent binding liability was uncovered with TAK-994 following hepatic metabolism consistent with idiosyncratic DILI and the delayed-onset clinical toxicity. Although idiosyncratic DILI is challenging to detect preclinically, reductions in total daily dose and covalent binding can reduce the covalent body binding burden and, subsequently, the clinical incidence of idiosyncratic DILI.

Impact statement We demonstrate that the likely mechanism of drug-induced liver injury (DILI) for TAK-994 is covalent-binding-dependent idiosyncratic DILI. Although many toxicities can be detected and derisked preclinically, there is room for improvement in both in vitro assays and in vivo studies as they have limited ability to predict and therefore protect patients from idiosyncratic DILI.

The preclinical evaluation of small-molecule safety has advanced significantly over the past 2 decades, resulting in improved regulatory recommendations for safety evaluation of small molecules before first-in-human clinical trials (e.g. International Council for Harmonization of Technical Requirements [ICH] M3, ICH S7A, and S7B). The impact of these changes on clinical adverse events (AEs) can be tracked by monitoring the emergence of serious AEs encountered by new small molecules entering phase I clinical trials. For example, drug-induced proarrhythmia was one of the most frequent severe AEs encountered in phase I clinical trials, affecting 5% of drugs reporting AEs from 2000 to 2004 (Redfern and Valentin 2011). The ICH S7B guidelines published in 2005 recommended testing new molecular entities in combination with in vitro and in vivo preclinical assays that detected the most common mechanisms of QT prolongation (Vargas et al. 2021). Since then, the frequency of novel small molecules encountering serious arrhythmias, long QT syndrome, or Torsade de Pointes in phase I has decreased by ∼70% (Fig. S1A) (Olson et al. 1998; Monticello et al. 2017; Clarivate 2022).

By contrast, drug-induced liver injury (DILI) has long been a leading cause of safety-related attrition in the clinic (Waring et al. 2015). Specifically, patients receiving a drug that causes hepatocellular injury with jaundice, without a significant obstructive component, are at particularly high risk of fatal DILI, a relationship known as Hy’s law (Temple 2006). Accordingly, a longstanding and widespread investment has been made to understand the mechanistic underpinnings of DILI, with PubMed cataloging >50,000 publications addressing “drug-induced liver injury” since 1945. These publications have contributed to the elucidation of many common mechanisms of liver toxicity, improved in vitro DILI assays and biomarkers. Despite these many advances, the standard nonclinical assays and endpoints required for preclinical DILI assessment have remained unchanged for over 2 decades. During this time, the frequency of severe DILI among new small molecules entering phase I clinical trials has also remained relatively unchanged (Fig. S1B and C), with notable phase II and III compounds terminated or received boxed warnings due to DILI in the past few years (e.g. tolebrutinib, atabecestat, MK-1942, and TAK-994) (Novak et al. 2020; Sanofi 2022; LaHucik 2023).

Advances in in vitro hepatocyte cultures, liver microtissues, and liver-on-a-chip technologies have enhanced our ability to detect direct injury to hepatocytes caused by intrinsic DILI (Proctor et al. 2017; Walker et al. 2020; Ewart et al. 2022). Intrinsic liver injury is characterized by rapid onset and can often be reproduced in preclinical species and in vitro assays. Although there is no formal definition of idiosyncratic DILI, it is typically characterized by an inability to reproduce the same type of injury in most animals, lack of injury in most treated patients, and delayed onset of ≥1 mo. Further, the histology of idiosyncratic DILI is very similar to that of viral hepatitis with a monocytic inflammatory infiltrate, and is often the clearest indicator of an immune-mediated mechanism. In some cases, there is a drug–haplotype association as a result of reactive metabolites formed during drug metabolism covalently binding cellular proteins that are presented by, or alter the function of, a specific HLA haplotype. Thus, the tissue damage caused directly by the drug is often minimal relative to the adaptive immune response to drug-modified proteins, often cytotoxic CD8+ T cells (Foureau et al. 2015). This interplay of hepatic metabolism and adaptive immune-mediated cytotoxicity is presumably why traditional cytotoxicity assays do not reliably predict the risk of idiosyncratic DILI (Uetrecht 2019).

TAK-994 was the first orally available novel small-molecule agonist of orexin receptor 2 (OX2R) to be evaluated in clinical trials. Both rat and nonhuman primate repeat-dose toxicity studies with TAK-994 failed to detect any hepatotoxicity liability before first-in-human studies (Ishikawa et al. 2023). Phase I clinical trials testing the safety and mean wakefulness time of healthy male volunteers taking TAK-994 on 2 consecutive days did not show any signs of hepatotoxicity (NCT04551079). In the TAK-994-1501 phase II clinical trial, 73 patients with narcolepsy type I were treated over the course of 8 wk with placebo or TAK-994 dosed at 30 mg twice daily, 90 mg twice daily, or 180 mg twice daily. TAK-994 effectively increased mean wakefulness time in people with narcolepsy (Dauvilliers et al. 2023), but further development was terminated due to hepatotoxicity. Herein, we discuss the strengths and limitations of current in vitro hepatotoxicity assays to detect intrinsic and idiosyncratic DILI, and assess their impact on a pharmaceutical pipeline.

## Materials and methods

Investigative experiments in hepatic cell culture systems examined cytotoxicity, mitochondrial toxicity, impact on drug transporter proteins, and covalent binding (CVB) with TAK-994. These experimental methods are briefly summarized below and detailed protocols can be found in the Materials and Methods S1.

To reduce the incidence of safety-related attrition in the small-molecule portfolio of a pharmaceutical company (Takeda Pharmaceuticals), a series of in vitro safety screening assays were implemented between 2017 and 2019, with an emphasis on reducing the risk of DILI. In addition, subsequent to the termination of TAK-994 phase II clinical trials, a series of in vitro assays were conducted to assess what, if any, known mechanisms of intrinsic DILI may be involved in TAK-994 hepatotoxicity.

Covalent binding of TAK-994 was assessed by incubating [^14^C]TAK-994 with human hepatocytes. The CVB level was determined by relating the total amount of radioactivity (pmol equivalent) to the protein concentration in each sample. Assessment of TAK-994 inhibition potential for the bile salt efflux pump (BSEP) was assessed by incubating [^3^H(G)]taurocholic acid with TAK-994 (0 to 100 μM/l) or cyclosporin A after addition of 10 μl of membrane vesicles expressing BSEP or control membrane vesicles. Scintillation counts were assessed in two 5-min cycles.

Toxicology of TAK-994 was assessed using lnSphero’s 3D lnSight Human Liver models, based on N-acetyl-L-cysteine causality assay, glutathione depletion assay, and lipopolysaccharide (LPS)-sensitization assay. Hepatotoxicity was assessed in 7- and 14-day mono and co-culture models, in which cells were exposed to TAK-994 (0.1, 0.3, 1, 3.16, 10, 31.6, or 100 μM) for 7 days.

Cholyl-lysyl-fluorescein (CLF) efflux for TAK-994 was assessed using human hepatocytes implanted into PXB-mice. PXB cells were exposed to TAK-994 (0.3, 1, 3, 10, 30, and 100 μM) or vehicle for 2 h and then incubated for an additional 30 min with TAK-994 or vehicle containing 5 μM CLF. CLF efflux and its inhibition by TAK-994 were assessed by fluorescence analysis.

Cytotoxicity of TAK-994 was evaluated in glucose or galactose medium after 24 h and in glucose medium after 72 h in HepG2 cells, with cryopreserved HepG2 cells thawed and seeded onto assay plates at 5,000 cells/well in glucose or galactose medium for the 24-h assay and at 2,500 cells/well in glucose medium for the 72-h assay. After ∼24 h of incubation at 37 °C in 5% CO_2_, the cells were incubated with test solutions under the same conditions for 24 h for the plate with 5,000 cells/well or 72 h for the plate with 2,500 cells/well. Cytotoxicity of TAK-994 was also assessed using HepG2 cells with CYP3A4 overexpression using a lentivirus vector. HepG2/CYP3A4 cells were incubated in media containing TAK-994 (0.09 to 200 μM), positive control aflatoxin B1 (0.04 to 80 μM), or 1.0% DMSO (negative control). In both cytotoxicity assays, adenosine triphosphate (ATP) content was assessed after incubation using a commercial luminescence assay (CellTiter-Glo; Promega Co., Ltd, WI, United States).

Higher total daily dose levels (>100 mg) of drugs administered have long been associated with DILI risk (Chen et al. 2016; Martin et al. 2022). In 1999, it had been observed that idiosyncratic DILI may also have a threshold, 10 mg daily dose, below which it has rarely, if ever, been reported (Uetrecht 1999). To determine if this 10-mg threshold for idiosyncratic DILI still held true with modern chemistry, we evaluated a published dataset of 357 drugs in the U.S. Food & Drug Administration (2023) DILIrank dataset, 29 of which had published links to idiosyncratic DILI. We also evaluated the preclinical safety packages for the 29 drugs using a database of preclinical safety studies for marketed pharmaceuticals (Pharmapendium) to determine how effective these required studies are in detecting idiosyncratic DILI.

There are several established genetic risk factors that can predispose patients to DILI (Stephens et al. 2021; Daly 2023). We evaluated patients from the TAK-994 phase IIb clinical trial for any enrichment of established DILI genetic risk factors among the subjects discontinued due to increases in liver transaminases.

## Results

### Implementation of in vitro assays to reduce safety-related attrition

**Fig. 1.**
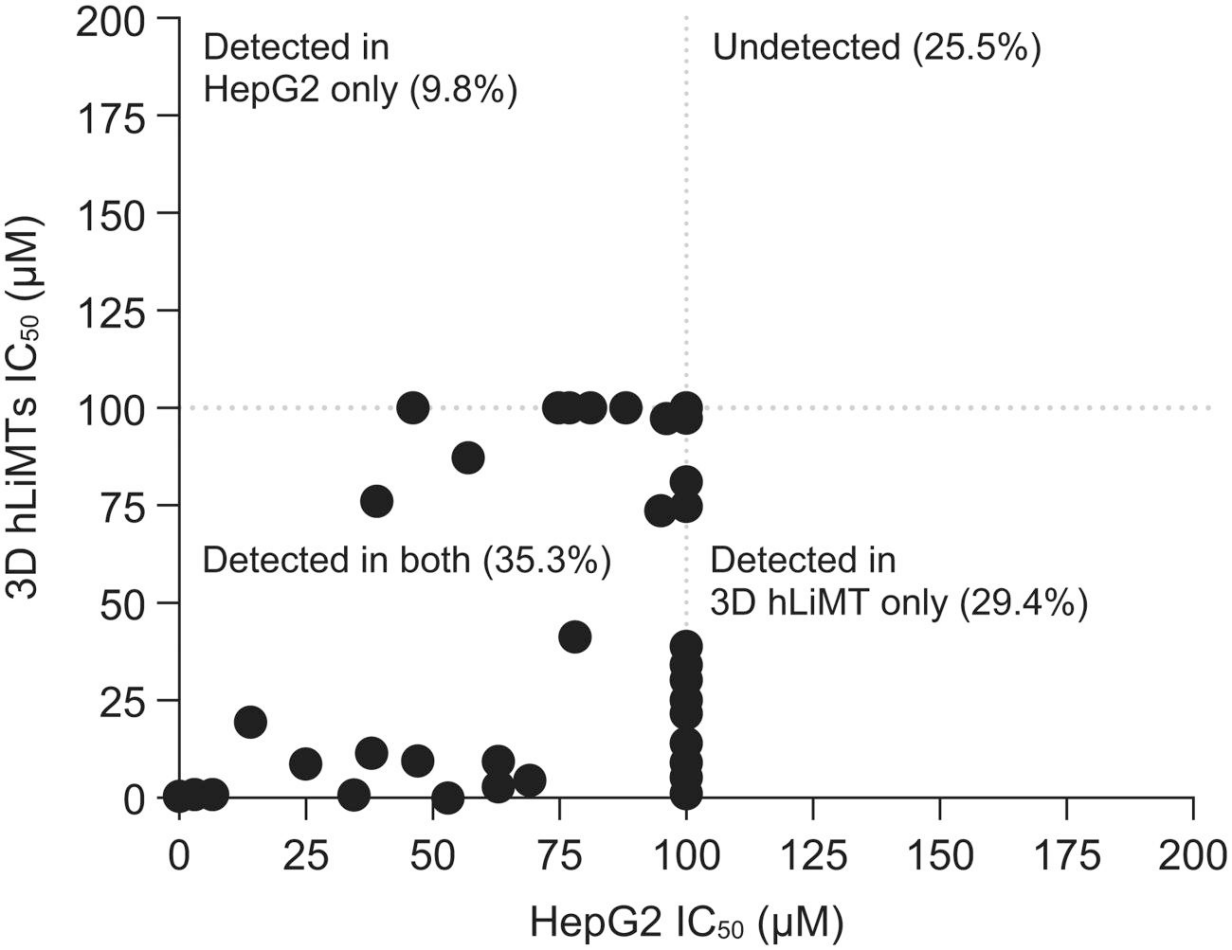
Preclinical compounds showing liver toxicity. Thirty-eight (74.5%) compounds from the preclinical pipeline were identified as having liver toxicity from assays in HepG2 or human liver microtissues (hLiMT), whereas 13 (25.5%) of compounds tested were undetected (shown at the midpoint on the graph). Eighteen (35.3%) compounds were detected in both assays, but only 5 (9.8%) and 15 (29.4%) compounds were detected in separate HepG2 and hLiMT assays. IC_50_, half-maximal inhibitory concentration.

Hepatotoxicity is a leading cause of safety-related attrition both preclinically and clinically (Waring et al. 2015). Two assays, a HepG2 72-h cytotoxicity assay and a human liver microtissue cytotoxicity assay, were retrospectively applied to 51 small-molecule drugs in Takeda’s pipeline that had been terminated due to direct/intrinsic liver toxicity between 2002 and 2017. These assays detected potential hepatotoxicity in 38 of 51 (74.5%) compounds, whereas 13 (25.5%) compounds were undetected in either assay (Fig. 1). Each of the 38 compounds was found to induce ≥50% cellular ATP depletion at or below 100 μM, a threshold commonly associated with hepatotoxic liability (Proctor et al. 2017). Importantly, 2 drug candidates stopped in phase III clinical trials due to hepatotoxicity, TAK-875 and TAK-475, were both detected using these assays (Table S1).

Application of these in vitro assays increases the quality of drug candidates going into good laboratory practice (GLP) toxicology studies and reduces the number of animal studies needed to support the drug discovery pipeline. However, the assays did not detect 29% of small molecules terminated due to preclinical hepatotoxicity findings, indicating that not all forms of DILI are readily detected preclinically with the current assays, as was the case for TAK-994.

### TAK-994 clinical and nonclinical liver evaluation

TAK-994 performed well in tiered safety screening assays, with wide margins between in vitro IC_50_ and the predicted efficacious exposures (Table 1). TAK-994 was well tolerated in nonclinical GLP toxicity studies in rats and cynomolgus monkeys, with no adverse liver findings up to the high dose tested of 1,000 mg/kg in 6- and 9-mo chronic dose toxicity studies in rats and cynomolgus monkeys, respectively (Table 2 and Table S2). Based on the safety profiles observed in these animal studies, TAK-994 progressed into phase I clinical trials.

**Table 1.** TAK-994 in vitro preclinical hepatic safety margins.

Assay		Criteria for reduced DILI risk	TAK-994 (BID)		
			30 mg	90 mg	180 mg
Clinical daily dose		Below 100 mg	60 mg	180 mg	360 mg
Cytotoxicity HepG2		IC_50_>50 μM	>100 µM	>100 µM	>100 µM
Mitochondrial toxicity in HepG2 (Glu/Gal)		Either no cytotoxicity (>100 μM) or Glu IC_50_/Gal IC_50_ <3-fold difference	>100 µM	>100 µM	>100 µM
Human liver microtissue		IC_50_/*C*_max_ margin >100×		100 µM	
			*C*_max_=245×	*C*_max_=105×	*C*_max_=61×
Bile transport assay (CLF) in hepatocytes		IC_50_/*C*_max_ margin >20×		30 µM	
			*C*_max_=74×	*C*_max_=32×	*C*_max_=18×
BSEP inhibition		IC_50_/*C*_max_ margin >10×	34.1 µM		
			83×	36×	21×
Daily CVB burden^a^		Below 1 mg	8.4 mg	25.1 mg	50.2 mg
Efficacious exposure in NT1 patients	*C*_max_	N/A	408 nM	948 nM	1,636 nM
	AUC_24, ss_		2,920 h*nM	8,870 h*nM	14,800 h*nM

All data and in vitro models were derived from humans.

a Reflects most conservative values based on internal assay data.

AUC_24_, area under the curve from 0 to 24 h; BID, twice daily; BSEP, bile salt efflux pump; CLF, cholyl-lys-fluorescein; *C*_max_, peak plasma concentration; CVB, covalent binding; DILI, drug-induced liver injury; Gal, galactose; Glu, glucose; IC_50_, half-maximal inhibitory concentration; NT1, narcolepsy type 1.

**Table 2.** TAK-994 preclinical hepatic safety evaluation in vivo.

Assay		Criteria for low DILI risk	TAK-994 (BID)		
			30 mg	90 mg	180 mg
In vivo exposure margin to NOAEL		Mouse	23×/9×	10×/3×	6×/2×
		Rat	75×/150×	32×/49×	19×/30×
		Nonhuman primate	132×/198×	57×/65×	33×/39×
Plasma exposures in NT1 patients	*C*_max_	N/A	408 nM	948 nM	1,636 nM
	AUC_24, ss_		2,920 h*nM	8,870 h*nM	14,800 h*nM

Reflects most conservative values based on internal assay data. Margins shown indicate fold difference between preclinical NOAEL and clinical *C*_max, total_. Mouse: *N* = 6 (3 female, 3 male); rat: *N* = 6 (3 female, 3 male); Nonhuman primate: *N* = 8 (4 female, 4 male).

AUC_24, ss_, area under the curve from 0 to 24 h, steady state; BID, twice daily; NOAEL, no observed adverse effect level; NT1, narcolepsy type 1.

TAK-994 was considered to be well tolerated in phase I studies. In the first-in-human study, TAK-994-1001, no deaths or SAEs were reported, and most AEs were consistent with the safety profile as predicted through preclinical studies. Of 121 participants, 1 subject discontinued due to elevated alanine aminotransferase (ALT) with no other liver enzyme elevations. This event was considered mild in intensity and the participant recovered within 2 wk. No other clinically significant liver enzyme elevations were reported in phase I studies.

Although phase I human clinical trials were ongoing, additional GLP-compliant repeat-dose oral toxicity studies were conducted in nonTg-rasH2 mice, to enable carcinogenicity studies. During these studies, hepatocellular injury was observed in TAK-994-treated mice. The liver toxicity presented at high doses ≥300 mg/kg/day as single-cell necrosis of periportal hepatocytes associated with increased aspartate aminotransferase (AST), alkaline phosphatase, ALT, and glutamate dehydrogenase values that increased in a dose-proportional manner alongside cytochrome P450 induction (Table S2).

A safety signal related to hepatotoxicity was detected in phase II studies in patients with narcolepsy type 1 or type 2, and the clinical program was voluntarily discontinued by Takeda to ensure patient safety (Dauvilliers et al. 2023). A total of 8 subjects in the 90- and 180-mg BID treatment groups met protocol-defined discontinuation criteria due to increases in liver transaminases in the TAK-994-1501 study and the extension study, TAK-994-1504. Among the 8 cases, 3 progressed to satisfy Hy’s Law criteria after termination of the clinical program and were diagnosed as DILI (Temple 2006). Liver biopsies were performed, and the histopathology findings were consistent with hepatitis involving mononuclear cell infiltrates. All 3 cases were in the higher-dose groups of 90 and 180 mg and had a delayed-onset latency with the earliest case emerging after 50 days of dosing. However, the elevations progressed rapidly closer to Hy’s Law within 3 wk of the initial transaminase elevation. All 3 patients were extensively monitored; they did not have symptoms related to hepatic impairment and have not required medical intervention for the treatment of hepatotoxicity. No other confounding or risk factors were identified. The first Hy’s Law case completely resolved in 55 days. The remaining 2 Hy’s Law cases have had a more prolonged recovery. Although their AST/ALT levels have normalized; their total bilirubin levels remain slightly elevated above the upper limit of normal. One of these cases was reported to be resolved after ∼2 yr since the elevations were first observed, whereas periodic monitoring will continue until resolution for the last case.

### Pharmacological hypothesis

Investigations into orexin receptor 1 (OX1R) and OX2R expression levels in human tissues found that neither receptor is detected in hepatic or immune tissues across Genotype-Tissue Expression or Human Protein Atlas databases (Fig. S2) (Uhlén et al. 2015). Additionally, mice with the orexin neuropeptide knocked out have no reported issues with hepatic or immune function (Chemelli et al. 1999). These data, together with the delayed-onset observed with TAK-994-associated DILI, indicate that orexin receptors are unlikely to directly play a role in the observed hepatotoxicity.

### Preclinical measures of intrinsic hepatotoxicity

**Fig. 2.**
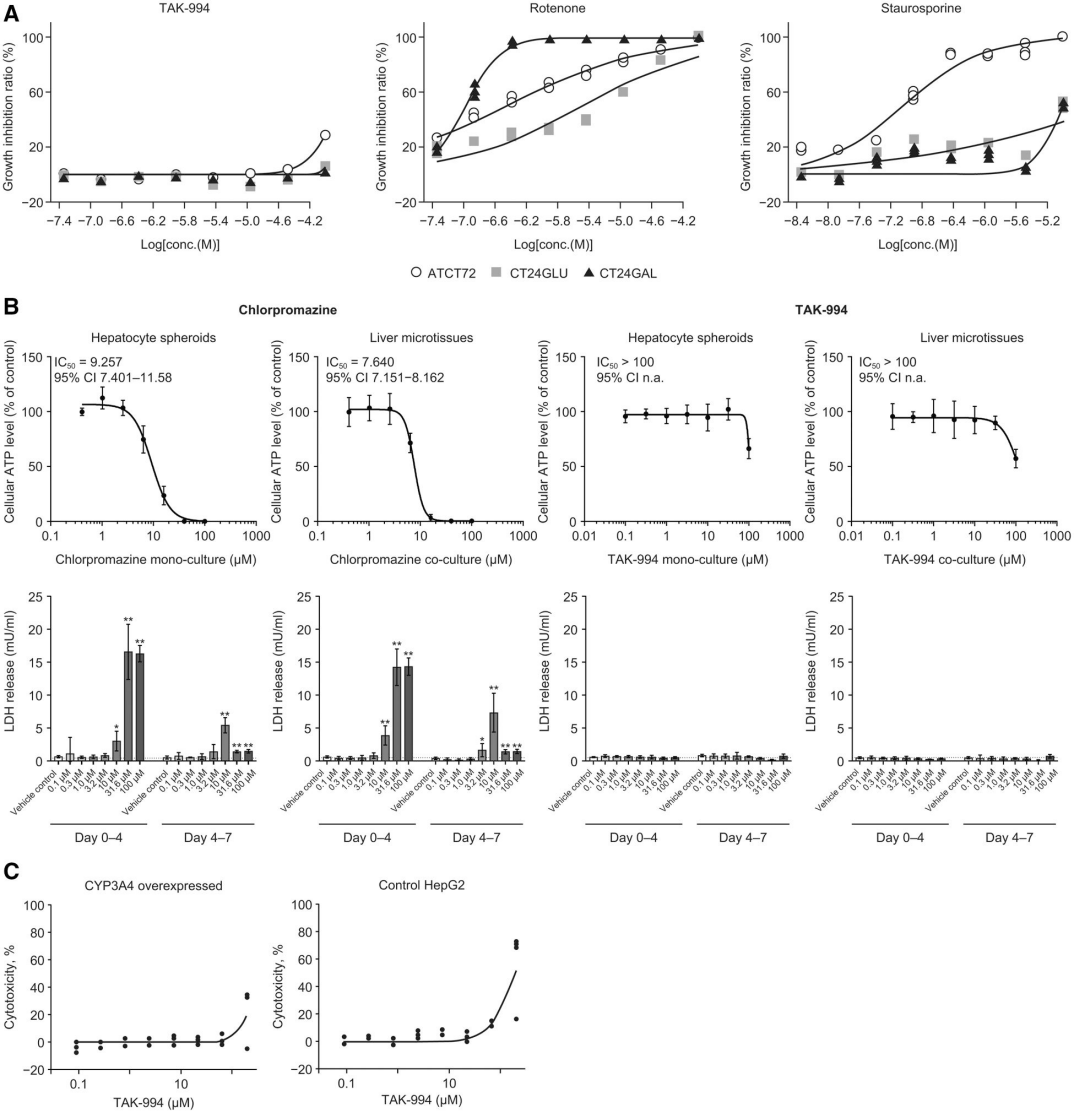
TAK-994 preclinical cytotoxicity studies. (A) TAK-994 did not cause HepG2 cytotoxicity or mitochondrial toxicity. Positive controls were rotenone and staurosporine; *n* = 2. ATCT72, 72 h in glucose medium; CT24GLU or CT24GAL, 24 h in glucose or galactose medium, respectively. (B) TAK-994 liver microtissue toxicity acts directly on hepatocytes. (C) TAK-994 treatment for 24 h showed no cytotoxicity difference between control HepG2 and CYP3A4 overexpressed cells; *n* = 3. Unpaired, 2-tailed *t*-test, *P ≤ 0.05; **P ≤ 0.01 vs vehicle control. IC_50_, half-maximal inhibitory concentration.

Cytotoxicity experiments demonstrated no detectable TAK-994-induced cytotoxicity on HepG2 cells under glycolytic conditions, or under growth conditions that rely on mitochondrial respiration for cell growth and proliferation up to 100 µM TAK-994, which is ∼100-fold higher than the peak plasma concentration (*C*_max_) in patients administered 90-mg doses (Fig. 2A). To determine whether TAK-994-mediated effects on hepatocytes are direct or indirect, human liver microtissues were cultured in the presence and absence of nonparenchymal cells. Hepatocytes under both conditions lost ∼50% of their cellular ATP at 100 µM TAK-994, suggesting that the in vitro effects of TAK-994 on hepatocytes could be direct (Fig. 2B). Assessment of HepG2 media acidification and oxygen consumption rate via Seahorse instrumentation similarly showed no TAK-994-induced changes to mitochondrial respiration. Similarly, HepG2 cells overexpressing cytochrome P450 (CYP) 3A4, which is the principal phase I enzyme responsible for TAK-994 metabolism, were not sensitive to TAK-994, indicating that CYP3A4 metabolites alone do not induce cytotoxicity in vitro (Fig. 2C).

Culture of liver microtissues with TAK-994 for 14 days induced a mild toxicity with an IC_50_ of ∼100 µM, which is >100-fold higher than the clinical *C*_max_ in patients dosed at 90 mg, where DILI was first observed.

**Fig. 3.**
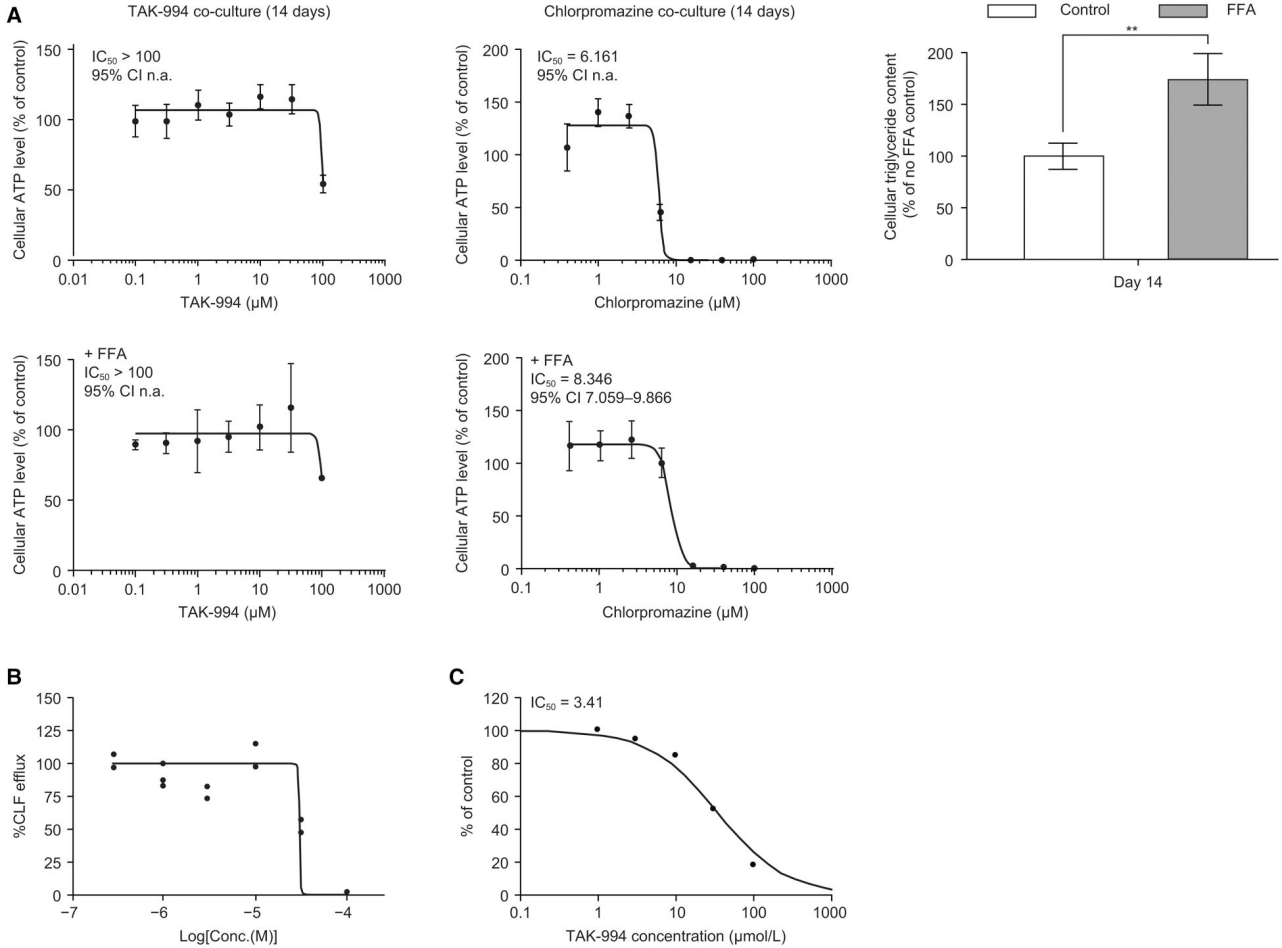
TAK-994 preclinical cytotoxicity studies. (A) TAK-994 liver microtissue toxicity not sensitized by intracellular triglycerides. Right, triglycerides measured in free fatty acid (FFA) models (Day 14). Unpaired, 2-tailed *t*-test, *P ≤ 0.05; **P ≤ 0.01 vs vehicle control. (B) Effect of cholyl-lys-fluorescein (CLF) efflux change after treatment with TAK-994; *n* = 3; IC_50_=30.0 μM. (C) TAK-994 inhibits bile salt efflux pump (BSEP)-mediated transport of [3H]TCA. [3H]TCA, [3H(G)]taurocholic acid; IC_50_, half-maximal inhibitory concentration.

**Fig. 4.**
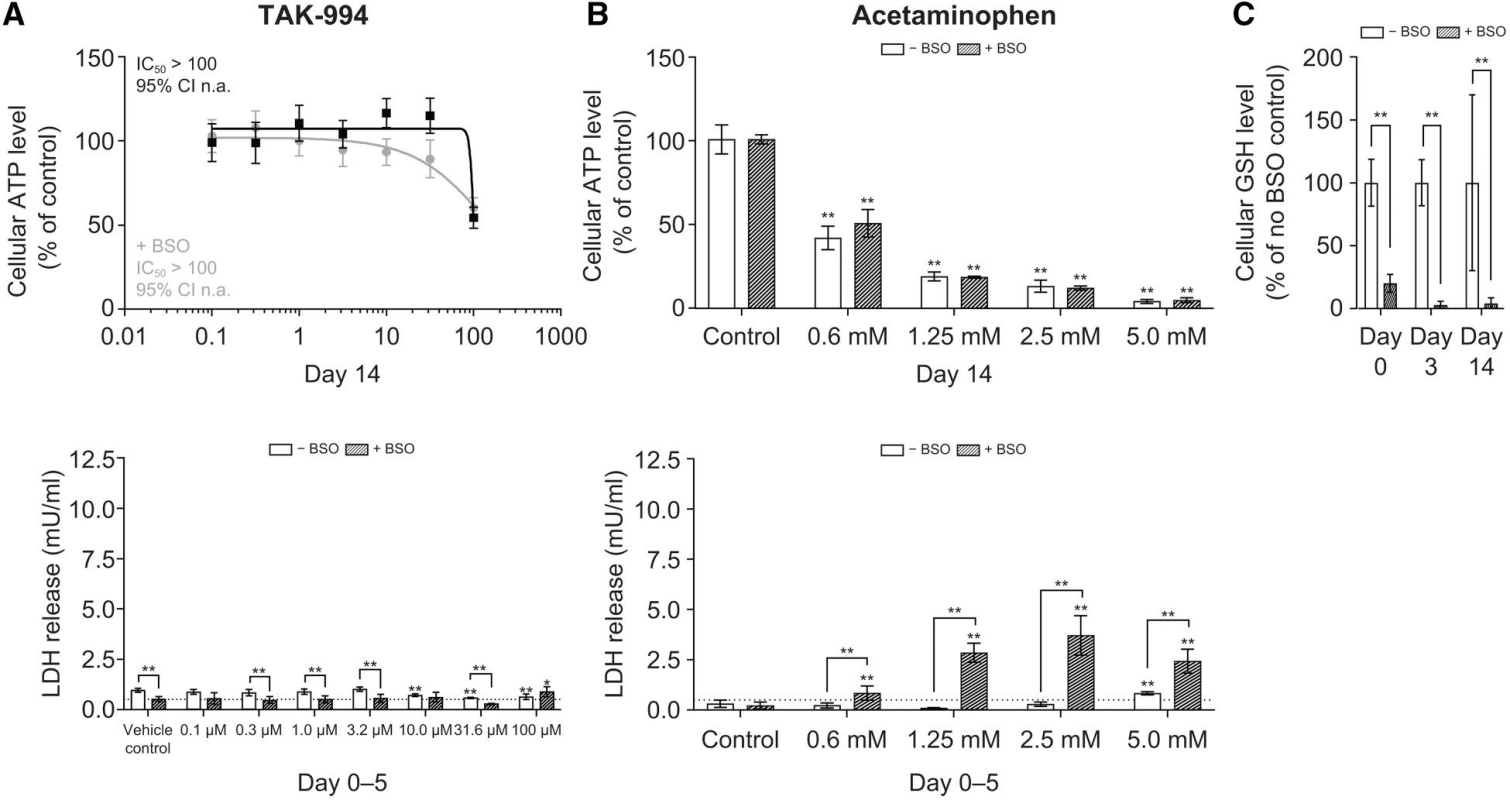
TAK-994 liver microtissue toxicity is not sensitized by BSO. The profile of cytotoxic response of (A) TAK-994 and (B) acetaminophen monitored by cellular ATP levels induced by treatment (top) and release of LDH between Days 0 and 5 of treatment (bottom). ATP data shown as mean and SD. LDH data shown as mean and 95% confidence interval, values below 0.5 mU/ml considered as background (bottom dotted line). (C) Cellular GSH is measured in liver microtissues pretreated with BSO and in control microtissues on Days 0, 3, and 14. Unpaired, 2-tailed *t*-test, *P ≤ 0.05; **P ≤ 0.01 vs vehicle control. Significance between 2 specific samples indicated using a bracket linking the 2 samples. ATP, adenosine triphosphate; BSO, buthionine sulfoximine; GSH, glutathione; LDH, lactate dehydrogenase.

Studies in liver microtissues showed that, although the addition of free fatty acids to liver microtissue media did increase fatty acid content of the liver microtissues, no increased sensitivity to TAK-994 was observed (Fig. 3A). Furthermore, TAK-994 inhibited the transport of fluorescently labeled bile salts (CLF) in human hepatocytes (Kohara et al. 2020) with an IC_50_ of 30.0 µM (Fig. 3B). This is likely due to the inhibition of the BSEP, where TAK-994 showed the inhibition with an IC_50_ of 34.1 µM (Fig. 3C). This inhibition is outside the 10-fold margin between IC_50_ and *C*_ss, avg_ recommended by the International Transporter Consortium (Kenna et al. 2018), whereas the clinical presentation of the hepatic injury was not consistent with cholestasis, suggesting that the BSEP is likely not driving the hepatotoxicity (Fig. 3C). To determine whether the effects of TAK-994 metabolites on hepatocytes could be due to reactive oxygen species-induced stress, cellular glutathione levels were depleted in liver microtissues with buthionine sulfoximine before treatment with TAK-994. Although buthionine sulfoximine depletion induced liver microtissue sensitivity to acetaminophen, which is known to induce reactive oxygen species in hepatocytes, no such sensitivity was observed with TAK-994 (Fig. 4).

**Fig 5.**
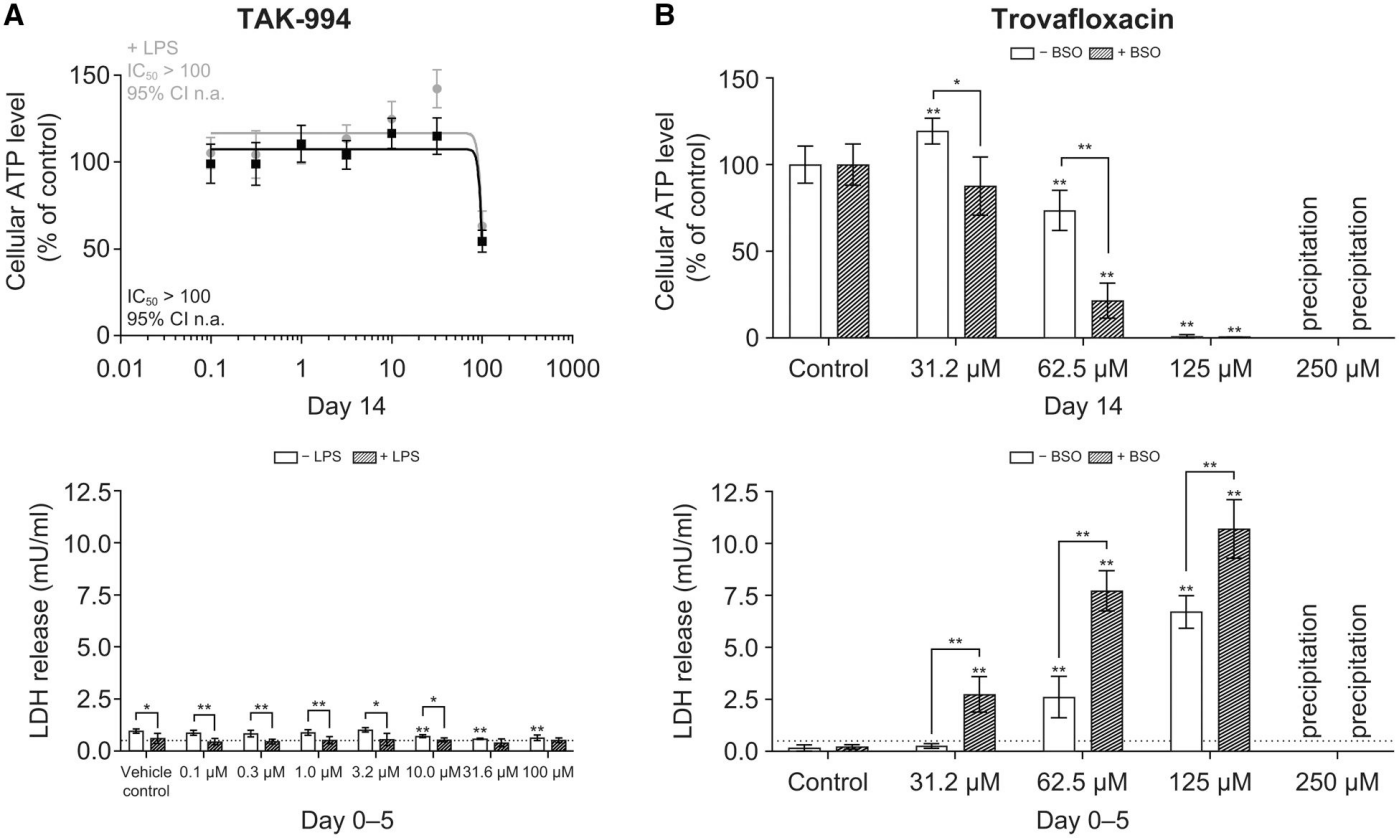
Cytotoxicity of TAK-994 in the presence and absence of LPS. The profile of cytotoxic response of (A) TAK-994 and (B) trovafloxacin as a positive control was monitored by release of LDH between Days 0 and 5 of treatment (top) and cellular ATP levels on Day 14 of treatment (bottom). LDH data shown as mean and 95% confidence interval, values below 0.5 mU/ml considered as background (bottom dotted line). ATP data shown as mean and SD. Unpaired, 2-tailed *t*-test, *P ≤ 0.05; **P ≤ 0.01 vs vehicle control. Significance between 2 specific samples is indicated by using a bracket linking the 2 samples. ATP, adenosine triphosphate; LDH, lactate dehydrogenase; LPS, lipopolysaccharide.

To investigate immune system activation, liver microtissues were treated with LPS to induce innate immune activation, which drives hepatotoxicity through an LPS-sensitive mechanism (Shaw et al. 2007). Although LPS sensitized liver microtissues to trovafloxacin-induced toxicity, no direct innate immune-mediated toxicity was observed with TAK-994 treatment (Fig. 5).

### Metabolism

To determine if reactive metabolites and covalent neoantigen formation could be contributing to TAK-994 DILI, we assessed TAK-994 for CVB potential relative to literature thresholds. TAK-994 was metabolically eliminated in human hepatocytes, and the CVB and daily CVB burden were assessed after 2-h incubation of human hepatocytes with TAK-994 at 10 μM. Under these conditions, the CVB level was determined to be 389.2 pmol equivalent/mg protein. The daily CVB burden was calculated assuming 100% of TAK-994 would undergo hepatic metabolism following previously published methodologies, resulting in estimated daily CVB values of 2.94 to 17.6 mg/day for clinical daily doses of 30 to 180 mg BID, which is above the published threshold of CVB that is associated with idiosyncratic DILI (Table 3) (Thompson et al. 2012). Notably, high levels of CVB alone are not sufficient to elicit DILI, as there are multiple examples of marketed drugs with elevated CVB without high risk of hepatotoxicity (e.g. ibuprofen and rimonabant) (Thompson et al. 2012).

**Table 3.** Estimated daily covalent binding burden in TAK-994 patients.

Daily dose (mg)	*f*_a_	*f*_m_	*f*_CVB_	CVB burden (mg)
60 (30 BID)	1^a^	1^a^	0.0490	2.94
180 (90 BID)				8.82
240 (120 BID)				11.8
360 (180 BID)				17.6

a *f*_a_ and *f*_m_ are assumed to be 1 in this calculation following worst-case scenario.

*f*_CVB_=[CVB×(protein concentration/well)]/[turnover×substrate concentration pmol/well].

CVB burden=dose×*f*_a_×*f*_m_×*f*_CVB_.

BID, twice daily; CVB, covalent binding, *f*_a_, fraction absorbed; *f*_CVB_, fraction of metabolism leading to covalent binding; *f*_m_, fraction of metabolism.

Based on the FDA DILIrank dataset, none of the 29 drugs with published links to idiosyncratic DILI had hepatic AEs published below 10 mg daily dose as a monotherapy (Fig. S3) and only 6 had any mention of a preclinical hepatotoxicity in their new drug applications (NDAs). Accordingly, we found that only 8 of the 29 drugs (27%) associated with idiosyncratic DILI had any clinical signs of hepatotoxicity at the time of NDA submission.

No link between discontinuation from the TAK-994 phase IIb clinical trial and established DILI genetic risk factors was found. We also found no evidence linking functional genetic markers in TAK-994 metabolizer genes to DILI risk in the TAK-994 phase IIb cohort (data not shown); details of analyses are provided in Materials and Methods S1.

## Discussion

The inclusion of in vitro assays as a part of an integrated preclinical safety screening strategy can help improve safety-related attrition for many, but not all, hazards. Since the release of ICH S7 guidelines mandating in vitro hERG patch clamp and in vivo nonrodent telemetry for the assessment of proarrhythmia before first-in-human studies for investigational drugs, the rate of serious arrhythmia reported in phase I clinical trials has fallen by ∼70% from 2000 to 2021 (Fig. S1A).

The success of this safety strategy is due in part to the presence of a common mechanism of proarrhythmia, hERG inhibition, which can be readily assessed with in vitro screens. DILI, however, remains a persistent challenge for new medicines entering phase I clinical trials—with no decrease in the incidence or severity of hepatobiliary disorders over that same time period (Fig. S1B and C). This finding was surprising given the breadth and depth of increased understanding of DILI mechanisms and predictive screening assays that have emerged over the past 2 decades. Retrospective analysis of 51 Takeda pipeline compounds that induced hepatotoxicity in vivo with 2 such assays (a HepG2 72-h cytotoxicity assay and a human liver microtissue cytotoxicity assay) found that 74.5% of the compounds induced cellular ATP depletion at or below the threshold commonly associated with hepatotoxic liability.

TAK-994, however, did not reach the threshold of concern on any of our routine safety screens. Similarly, the 26-wk rat and 39-wk non-human primate repeat-dose toxicology studies that were used to identify target organs & first-in-human dose levels did not identify the liver as a target organ of concern. These “false-negative” results are emblematic of the challenge facing the preclinical detection of idiosyncratic DILI. Only 6 (21%) of the 29 compounds clinically associated with idiosyncratic DILI detected hepatotoxic liability in their preclinical safety package, and only 8 (27%) of those compounds encountered hepatobiliary concerns before filing their NDA. Although TAK-994 was in clinical trials, 4-wk mouse studies were conducted in nontransgenic littermates from Tg.rasH2 mice colonies in preparation for NDA-enabling carcinogenicity studies. It was in these studies that periportal single-cell hepatic necrosis was noted along with CYP3A4 induction and concomitant liver injury biomarker elevations in clinical chemistry. These results in the nontransgenic littermates of Tg.rasH2 mice were taken in the context of phase 1 clinical trials, 26-wk rat studies and 39-wk nonhuman primate studies in which no hepatotoxic findings had been observed. Given the high levels of oxidative defluorination and CYP-induction observed in the mice, it is believed that high levels of free fluorine could be contributing to the single-cell necrosis. No immune infiltration or inflammation was noted in the livers of the affected mice.

Idiosyncratic DILI is often immune-mediated (Jee et al. 2021). One of the most common mechanisms by which immune-mediated liver toxicity occurs is through the metabolism of parent drug, resulting in reactive metabolites that bind endogenous proteins, creating neoantigens that elicit an adaptive immune response (Cho and Uetrecht 2017). The formation of metabolism-induced CVB can be modeled in vitro. Although different methodologies have been employed and have arrived at different thresholds for idiosyncratic DILI concern, one of the most widely cited is a threshold of 1 mg daily CVB burden, below which idiosyncratic DILI has not been reported (Bauman et al. 2009; Nakayama et al. 2009; Thompson et al. 2012; Kakutani et al. 2019). Small-molecule drugs with an estimated CVB burden above 1 mg have been associated with higher concern of idiosyncratic DILI, especially when those drugs inhibit BSEP, cause cytotoxicity, mitochondrial toxicity, or impact other established mechanisms for intrinsic DILI (Thompson et al. 2012; Walker et al. 2020).

Although these in vitro mechanistic assays and considerations around daily dose and CVB have been linked to DILI in the literature, they are not commonly used for clinical dose setting or required for the preclinical safety evaluation of small molecules. Evaluation of investigative new drugs for liver toxicity occurs as part of the repeat-dose toxicology studies in 2 mammalian species (1 nonrodent) required by ICH M3 and S9 guidelines for the evaluation of small molecules before first-in-human dosing, generally clinical pathology, organ weight, and macro/microscopic pathology.

Idiosyncratic DILI is often the result of multiple independent and interdependent pathways, frequently resulting in a low incidence rate, with many drugs of iDILI concern inducing no clinical signs of DILI until after 10,000 to 100,000 patient-years (Fontana 2014). In those cases in which the pharmacological target plays a role in DILI, the target has always been expressed on hepatic cells, immune cells, or both (Uhlén et al. 2015; Hoofnagle and Björnsson 2019). In the case of orexin receptor agonists, however, the Human Protein Atlas indicates that neither OX1R nor OX2R are expressed on human hepatic or immune cells (Fig. S2) (Uhlén et al. 2015, 2019).

Although it is plausible that BSEP inhibition could be a risk factor for idiosyncratic DILI, there is little direct evidence to support the hypothesis. Very few studies have been done to measure serum bile acids in patients treated with drugs that inhibit BSEP. Published work has shown that BSEP inhibition is not a good predictor of idiosyncratic DILI risk (Chan and Benet 2018). Although TAK-994 did inhibit BSEP in vitro, this inhibition occurred at concentrations outside the threshold of concern recommended by the International Transporter Consortium (>10 × IC_50_/*C*_ss, avg_). In our clinical studies, the bile acid changes only occurred after prolonged transaminase elevation, suggesting BSEP inhibition is less likely to be one of the driving factors in the observed idiosyncratic DILI.

The inflammagen model of DILI, or co-treatment of LPS with TAK-994, is not a direct test of idiosyncratic DILI potential. Instead, it tests whether simultaneous innate immune stimulation could sensitize hepatocytes to TAK-994, as they do to trovafloxacin. LPS, however, did not have any effect on TAK-994-induced toxicity to liver microtissues in vitro.

There is strong evidence that inhibition of the mitochondrial electron transport chain is not involved in the mechanism of idiosyncratic DILI (Cho et al. 2019). Metformin, which is a potent inhibitor of the mitochondrial electron transport chain and causes lactic acidosis rarely, if ever, causes idiosyncratic DILI. Inhibition of the mitochondrial electron transport chain might be insufficient by itself to cause idiosyncratic DILI, however, metformin does not increase the risk of co-administered drugs. There is strong evidence that valproic acid-dependent idiosyncratic DILI involves mitochondria, but not inhibition of the electron transport chain, and the characteristics of valproate idiosyncratic DILI are quite different and clearly show mitochondrial involvement. Other DILI, such as caused by fialuridine, clearly involves mitochondria but it is not idiosyncratic and is not limited to the liver (McKenzie et al. 1995).

Takeda’s working hypothesis for TAK-994-related DILI is that reactive intermediates transiently formed during the oxidative metabolism of TAK-994 and produced a persistent low-grade hepatic insult and elevated plasma transaminase levels. The CVB of these reactive intermediates resulted in formation of protein adducts that served as neoantigens which, in the most severe cases, led to the development of delayed-onset idiosyncratic DILI. This covalent-binding burden was detected retroactively and determined to be above previously published thresholds of concern. Reducing reactive metabolite formation and covalent body burden is associated with lower levels of idiosyncratic drug reactions, and deprioritizing drugs that are likely to cause high levels of CVB can help to reduce the risk of idiosyncratic DILI in the clinic.

That said, the prediction of immune AEs is difficult due to the differences in immune responses between patients as well as the profound differences in responses between species. The scale of challenge presented by predicting immune responses across patients and species is in part why the frequency of immune-related AEs reported to the FDA AEs database has risen over 10-fold in the past 20 yr.

## Conclusions

Together, these clinical, in vitro, and in vivo data point to a gap in our ability to detect and derisk idiosyncratic DILI before new therapeutic agents reach patients. Detailed understanding of the mechanisms of idiosyncratic DILI is aiding the development of improved idiosyncratic DILI detection assays (Kato and Uetrecht 2017; Kato et al. 2019, 2020, 2021, 2023; Kato and Ijiri 2022; Sernoskie et al. 2022), which rely on modeling the early stages of innate immune response that is required to get a strong adaptive immune response. Although these assays have shown great promise in early studies, additional work is needed to determine their suitability for preclinical safety screening. Although clinical risk of idiosyncratic DILI may never be fully eliminated preclinically, reductions in covalent-binding potential, total daily dose, and intrinsic DILI risk factors can decrease the risk of idiosyncratic DILI today, as improved DILI assays and strategies are being developed.

## Supplementary Material

kfaf003_Supplementary_Data

## Data Availability

The datasets generated during and/or analyzed during the current study are available from the corresponding author on reasonable request.
