# Supplementary material for: TAK-994 mechanistic investigation into drug-induced liver injury
Source: Toxicol Sci. 2025 Jan 9;204(2):143–53. doi: 10.1093/toxsci/kfaf003 (PMC11939078; doi:10.1093/toxsci/kfaf003)
Supplement: kfaf003_Supplementary_Data [file kfaf003_supplementary_data.docx]

**TAK-994 Mechanistic Investigation into Drug-Induced Liver Injury**

Tadahiro Shinozawa, Kazumasa Miyamoto, Kevin S. Baker, Samantha C. Faber, Ramon Flores, Jack Uterecht, Christian von Hehn, Tomoya Yukawa, Kimio Tohyama, Harisha Kadali, Marcin von Grotthuss, Yusuke Sudo, Erin N. Smith, Dorothée Diogo, Andy Z. X. Zhu, Yvonne Dragan, Gvido Cebers, Matthew P. Wagoner

**Supplementary Material**

**Table of contents**

**Supplementary Materials and Methods 3**

1. *Covalent binding assessment of [^14^C]TAK-994* *3*
2. *Assessment of TAK-994 inhibition potential for BSEP 4*
3. *Investigative toxicology study of TAK-994 (TR04590394) using lnSphero's 3D lnSight^TM^ Human Liver models* *5*
4. *CLF efflux assay for TAK-994 using PXB cells 8*
5. *Cytotoxicity of TAK-994 in glucose or galactose medium after 24 hours and in glucose medium after 72 hours in HepG2 cells 10*
6. *Cytotoxicity assay for TAK-994 using CYP3A4 overexpressed HepG2 cells 11*
7. *Evaluation of ETC parameters using the Mito Stress Test 12*
8. *Genetic data generation and analysis 12*

**Supplementary Tables and Figures 14**

Supplementary Table 1 14

Supplementary Table 2 15

Supplementary Figure 1 17

Supplementary Figure 2 19

Supplementary Figure 3 20

**References 21**

Supplementary Materials and Methods

1. *Covalent-binding assessment of [^14^C]TAK-994*

[^14^C ]TAK-994 (10 μmol/L, supplied by Takeda Pharmaceutical Company Limited, Japan) was incubated with human hepatocytes (LIVERPOOL^®^ 10-donor mixed gender, pooled, cryopreserved human hepatocytes [Lot No. ZOL, BioIVT]) as suspension culture in 24-well plates at 37°C under 5% CO_2_, humidified, for 0 or 2 hours. Seeding density of the cells was 3.0 × 10^5^ viable cells/500 μL/well. A total of 3 replicates were assessed.

The covalent-binding (CVB) level was determined by relating the total amount of radioactivity (pmol equivalent) to the protein concentration in each sample. To obtain the net covalent-binding (CVB*net*), the average result of the “back-added” samples (CVB*back*) was subtracted from the average result of the incubated samples (CVB*_inc_*). For the determination of [^14^C ]TAK-994 turnover in the incubation, part of the samples were analyzed by high-performance liquid chromatography with an online flow scintillation analyzer.

The turnover of TAK-994 was calculated using the following equation:

Turnover = 1 − [percentage of TAK-994 relative to the total radioactivity at 2 hours]
[percentage of TAK-994 relative to the total radioactivity at 0 hour]

The fraction of metabolism leading to covalent binding (f*_cvb_*) was calculated as follows:

f*_cvb_* = CVB (pmol/mg protein) × [mean protein concentration (mg/well)]
turnover × [substrate concentration (pmol/well)]

The CVB burden^1^ was calculated using the equation below:

CVB burden (mg) = *D* × *f_a_* × *f_m_* × *f_cvb_*

*D*: prescribed daily dose (mg)
*f_a_*: fraction of the dose absorbed
*f_m_*: fraction of the dose eliminated via metabolism

*D* was set to 60, 180, 240, and 360 mg. Both *f_a_* and *f_m_* were set to a value of 1 (Thompson et al. 2012).

1. *Assessment of TAK-994 inhibition potential for BSEP*

Chemicals and reagents:

TAK-994 was supplied by Takeda. Radiolabeled substrate [^3^H(G)]taurocholic acid ([^3^H]TCA; 37 MBq/mL) was purchased from PerkinElmer (Waltham, MA, USA). Unlabeled substrate sodium taurocholate (TCA) was purchased from FUJIFILM Wako Pure Chemical Corporation (Osaka, Japan). Reagents used in this study were purchased from commercial suppliers and were of analytical grade or equivalent.

Membrane vesicles:

Membrane vesicles prepared from baculovirus-infected insect cells, expressing human BSEP or controls (Lot Nos. ISAOG29 and IKING23, respectively), were purchased from GenoMembrane Co., Ltd.

Investigation of the inhibitory effect of TAK-994 on the uptake of [^3^H]TCA into membrane vesicles expressing BSEP:

The [^3^H]TCA reaction mixture (n=42) containing TAK-994 (including 0–100 μM/L TAK-994) or cyclosporin A was pre-incubated at 37°C for 5 minutes. The transport reaction was initiated by addition of 10 μL of membrane vesicles expressing BSEP or control membrane vesicles (0.05 mg protein) to the [^3^H]TCA reaction mixture (final [^3^H]TCA concentration of 2 μM/L). The reaction mixture was incubated with shaking at 37°C for 2 minutes and then terminated by adding 1 mL of ice-cold stopping buffer. Immediately after termination of the reaction, an aliquot (900 μL) of the stopped reaction mixture was filtrated through a glass fiber filter and then washed twice with 10 mL of ice-cold stopping buffer. The glass fiber filter was measured for radioactivity using an LSC. All incubations were made in triplicate.

Scintillation counting:

The count rates in cpm were corrected to dpm, with the background and counting efficiency assessed using the external standard method described by the manufacturer. The measurement time was set at 5 minutes, and the measurement was performed in 2 cycles. The values from the second cycle were adopted.

1. *Investigative toxicology study of TAK-994 (TR04590394) using lnSphero's 3D lnSight^TM^ Human Liver models*

Chemicals and reagents:

TAK-994 was supplied by Takeda. Vehicle control: 0.5% dimethyl sulfoxide (DMSO). Positive controls: acetaminophen (Catalogue No. A7085, Sigma Aldrich), menadion (Catalogue No. T0449, TargetMol), aflatoxin B1 (Catalogue No. HY-N6615, MedChemExpress), trovafloxacin (Catalogue No. PZ0015, Sigma Aldrich), and chlorpromazine (Catalogue No. 16129, Cayman). Other: N-acetyl-L-cysteine (NAC; Catalogue No. A9165, Sigma Aldrich), buthionine sulfoximine (Catalogue No. B2515, Sigma Aldrich), lipopolysaccharide (LPS) from *Escherichia coli* O55:B5 (Catalogue No. L2880, Sigma Aldrich).

Test system:

The test system was Human 3D InSight Liver Models, production ID hLiMT_589, cryopreserved primary human hepatocytes (Lot IPDH_18; single donor) in monoculture or in co-culture with cryopreserved primary human nonparenchymal cells (Lot IPHN_17; single donor). Human liver microtissues are composed of roughly 1000 hepatocytes and 1000 non-parenchymal live cells. Non-parenchymal liver cell fractions are expected to be composed of Kupffer cells and liver endothelial cells however, these were not identified.

Cell culture:

The culture medium was 3D InSight Human Liver Maintenance Medium TOX (Catalogue No. CS-07-001, InSphero). The treated cells in 96-well Akura™ plates were incubated in a humidified cell culture incubator (37°C, 5% CO_2_).

N-acetyl-L-cysteine (NAC) causality assay:

Co-culture model cells were pretreated with NAC and then exposed to TAK-994 (0.1, 0.3, 1, 3.16, 10, 31.6, or 100 μM in 0.5% DMSO) in the presence and absence of NAC for 14 days. During the treatment period, microtissues were exposed to TAK-994 with or without NAC on Days 0, 5, and 9. Six replicates were tested for each condition. Controls included vehicle control 0.5% DMSO and positive controls acetaminophen (0.4, 0.7, 1, 1.5, 2.2, 3.3, and 5 mM in Medium TOX) and menadione (1.56, 3.13, 6.25, 12.5, 25, 50, or 100 μM in DMSO). Adenosine triphosphate (ATP) content was measured in the cell lysate on Day 14 of treatment for IC_50_ calculation. LDH release was measured on Days 5, 9, and 14 in the cell supernatants and normalized to lysed controls.

Glutathione depletion assay:

Co-culture model cells were pretreated with BSO (150  μM in DMSO) and then exposed to TAK-994 (0.1, 0.3, 1, 3.16, 10, 31.6, or 100 μM in 0.5% DMSO) in the presence and absence of BSO for 14 days (during the treatment period, TAK-994 with or without BSO was administered on Days 0, 5, and 9). Six replicates were tested for each condition. The vehicle control was 0.5% DMSO and the positive control was acetaminophen (0.6, 1.25, 2.5, or 5 mM in Medium TOX). Cellular ATP content was measured in the cell lysate on Day 14 of treatment. LDH release was measured on Days 5, 9, and 14 in the cell supernatant. For cells administered BSO alone, cellular ATP content was also measured on Day 3 and LDH release was also determined between Days 0 to 3. LDH measurements were normalized to lysed controls. Cellular GSH content was measured by GSH-Glo™ Glutathione Assay (Promega) on Days 0, 3, and 14 in the positive control group.

LPS-sensitization assay:

Co-culture model cells were pretreated with LPS (5 ng/mL in phosphate-buffered saline) and then exposed to TAK-994 (0.1, 0.3, 1, 3.16, 10, 31.6, or 100 μM in 0.5% DMSO) in the presence and absence of LPS for 14 days (during the treatment period, TAK-994 with or without LPS was administered on Days 0, 5, and 9). Six replicates were tested for each condition. The vehicle control was 0.5% DMSO, and the positive control was trovafloxacin (31.25, 62.5, 125, or 250 mM in DMSO). Cellular ATP content was measured in the cell lysate on Day 14 of treatment. LDH release was measured on Days 5, 9, and 14 in the cell supernatant and normalized to lysed controls. Cellular GSH content was measured on Days 0, 3, and 14 in the positive control group treated with trovafloxacin.

Seven- and 14-day hepatotoxicity testing:

Mono and co-culture model cells were exposed to TAK-994 (0.1, 0.3, 1, 3.16, 10, 31.6, or 100 μM in 0.5% DMSO) for 7 days (TAK-994 administered on Days 0 and 4) or 14 days (TAK-994 administered on Days 0, 5, and 9). Six replicates were tested for each condition. The vehicle control was 0.5% DMSO, and the positive control was chlorpromazine (0.1, 0.3, 1, 3.16, 10, 31.6, or 100 μM in DMSO). Cellular ATP content was measured in the cell lysate on Days 7 or 14 of treatment. LDH release was measured in the cell supernatant on Days 4 and 7 or on Days 5, 9, and 14 and normalized to lysed controls.

Hepatotoxicity testing on 3D InSight™ human steatosis liver models:

Mono and co-culture model cells were exposed to TAK-994 (0.1, 0.3, 1, 3.16, 10, 31.6, or 100 μM in 0.5% DMSO) for 14 days (TAK-994 administered on Days 0, 5, and 9). The cell culture medium was supplemented with free fatty acids of 100 μM of Oleic acid and 66 μM of Palmitic acid. Six replicates were tested for each condition. The vehicle control was 0.5% DMSO, and the positive control was chlorpromazine (0.1, 0.3, 1, 3.16, 10, 31.6, or 100 μM in DMSO). Cellular ATP content was measured in the cell lysate on Day 14 of treatment. LDH release was measured in the cell supernatant on Days 5, 9, and 14 and normalized to lysed controls. Cellular triglyceride content was measured in the microtissues on Day 14.

Cellular assays:

Intracellular ATP content in model lysates was measured with the CellTiter-Glo^®^ 2.0 Cell Viability Assay (Catalogue No. G9243, Promega). The Bioluminescent LDH Release Toxicity Assay Kit (Catalogue No. J2380, Promega) was used to assess extracellular LDH release and normalized to lysed controls. ATP, LDH, GSH, and triglycerides were measured according to internal technical operating procedures.

1. *CLF efflux assay for TAK-994 using PXB-cells^®^ (Kohara et al. 2020)*

PXB hepatocytes were generated by implanting human hepatocytes into PXB-mice^®^. These hepatocytes were isolated by collagenase perfusion at PhoenixBio Co Ltd. Freshly isolated hepatocytes were maintained in T-75 flasks and detached using 0.25% trypsin/ethylenediaminetetraacetic acid (EDTA) incubation for 20 minutes (total) using the following procedure. A flash wash was performed with cold trypsin/EDTA, followed by a 5-minute incubation with cold trypsin/EDTA at 37°C, and finally a 15-minute incubation at 37°C with trypsin/EDTA. The cells were resuspended in hepatocyte clonal growth medium (dHCGM; PhoenixBio Co Ltd) and then replated at 7 × 10^4^ cells per well in collagen I-coated 96-well plates. The culture medium was replaced with fresh dHCGM after 4 hours. For all experiments, the culture medium was replaced with fresh Hepatocyte Culture Medium (HCM; Lonza) containing approximately 0.4 mg/mL Matrigel^®^ (Corning) on Days 1 and 4. On Day 6, the medium was replaced with fresh HCM. On Day 8, the cells were used in the cholyl-lysyl-fluorescein (CLF) efflux assay.

On Day 8, PXB cells were exposed to TAK-994 (0.3, 1, 3, 10, 30, and 100 μM) or 0.1% DMSO for 2 hours and then incubated for an additional 30 minutes with TAK-994 or 0.6% DMSO containing 5 μM CLF. The cells were washed 3 times using 150 μL, 100 μL, and finally 50 μL of fresh HCM medium per wash. Fluorescent images were captured using an IN Cell Analyzer 6500HS (GE Healthcare Life Sciences). The CLF efflux and its inhibition by TAK-994 was quantified using Developer toolbox software. High-content fluorescent cell images were captured using the device at multiple locations per well in a 96-well plate. The images were averaged for 16 locations per well. Fluorescence intensity in the presence of DMSO (vehicle control) was set as 0% inhibition of CLF efflux. The fluorescent intensities of bile canalicular regions were measured to determine the IC_50_ for TAK-994 CLF efflux inhibition. In general, fluorescence intensity in the presence of 3 μM cyclosporine was used as 100% inhibition of CLF. If the fluorescence intensity at 3 μM cyclosporine was higher than fluorescence intensity at the maximum concentration of test compounds, fluorescence intensity at the maximum concentration of TAK-994 was set as 100% inhibition.

1. *Cytotoxicity of TAK-994 in glucose or galactose medium after 24 hours and in glucose medium after 72 hours in HepG2 cells (Rana et al. 2019)*

HepG2 cells (American Type Culture Collection [ATCC, VA, USA]) were acclimated to Dulbecco's Modified Eagle Medium (DMEM; Thermo Fisher Scientific, MA, USA) with either high glucose or high galactose and containing 10% FBS for 1 week then cryopreserved. The cryopreserved HepG2 cells were thawed, cultured for 24 hours, then seeded at 5000 cells/well in glucose or galactose medium for the 24-hour assay and at 2500 cells/well in glucose medium for the 72-hour assay onto the assay plates. After approximately 24 hours of incubation at 37°C in 5% CO_2_, the cells were incubated with test solutions under the same conditions for 24 hours for the plate with 5000 cells/well or 72 hours for the plate with 2500 cells/well. After the incubation, CellTiter-Glo^®^ (Promega Co., Ltd., WI, USA) was added to the assay plates, cells were incubated for 30 minutes, and luminescence was recorded to measure the ATP content using an EnVision plate reader (PerkinElmer Co., Ltd., MA, USA). The test compound TAK-994 was evaluated at concentrations of 0.046, 0.14, 0.41, 1.2, 3.7, 11, 33, and 100 μM. Positive controls rotenone and staurosporine were purchased from FUJIFILM Wako Pure Chemical Corporation (Osaka, Japan) and evaluated at concentrations of 0.046, 0.14, 0.41, 1.2, 3.7, 11, 33, and 100 μM in DMSO (rotenone) or 0.0046, 0.014, 0.041, 0.12, 0.37, 1.1 , 3.3, and 10 μM in DMSO (staurosporine). Two experiments were conducted at each concentration.

The inhibitory rate of ATP content was calculated according to the following formula.

Inhibition rate (%) = (1-mean of sample values / mean of Max values) × 100, where Max values = vehicle control.

1. *Cytotoxicity assay for TAK-994 using CYP3A4 overexpressed HepG2 cells*

HepG2 cells were plated in a 10-cm dish and transduced with lentivirus (CYP expression viral package; MISSION^®^ Lentiviral Packaging Mix, Sigma Aldrich, St. Louis, MO, USA). After transduction, cells were grown in culture media containing blasticidin (final concentration of 10 μg/mL). HepG2 cells transduced with empty lentivirus were used for the negative control. The transduced HepG2 cells (HepG2/CYP3A4) were cultured for an additional week, refreshing periodically with culture media containing blasticidin. Cell pellets were collected for messenger RNA (mRNA) analysis, mycoplasma testing, and protein analysis. In addition, freeze-downs of live cells were made in 95% FBS + 5% DMSO.

HepG2/CYP3A4 cells were cultured in DMEM high glucose medium containing 2 mM L-glutamine, 1 mM sodium pyruvate, 5 mM 4-(2-hydroxyethyl)-1-piperazineethanesulfonic acid (HEPES), 10% FBS, 100 U/mL penicillin, and 100 μg/mL streptomycin. The cells were seeded at 2500 cells/20 μL/well on 384-well plates (Greiner, Kremsmünster, Austria) and incubated for approximately 18 hours at 37°C in 5% CO_2_. After incubation, media was changed to DMEM containing 0.5% FBS and 20 μL of the media containing TAK-994 or positive control aflatoxin B1 (FUJIFILM Wako Pure Chemical Corporation). Concentrations evaluated were 0.09 to 200 μM TAK-994 in DMSO, 0.04 through 80 μM aflatoxin B1 in DMSO, and 1.0% DMSO (negative control). The plate was incubated for 24 hours at 37°C in 5% CO_2_. Subsequently, 20 μL of CellTiter-Glo^®^ (Promega, Madison, WI, USA) reagent was added to each well, and the luminescence was measured using an EnVision plate reader (PerkinElmer Co., Ltd. [Waltham, MA, USA]) to quantify the adenosine triphosphate (ATP) in the metabolically active cells. The values of the 0% and 100% inhibition controls were obtained from wells containing cells incubated with DMSO and the medium only, respectively.

IC_50_ values were calculated by fitting a sigmoidal dose-response curve to the plot of the percentage of inhibition as a function of inhibitor concentration. Statistical analysis and fittings were performed using the XLfit software (IDBS, Woking, UK).

1. *Evaluation of ETC parameters using the Mito Stress Test*

The test was conducted using HepaRG cells (HPR116, Biopredic International, Saint Grégoire, France). The extracellular acidification rate (ECAR) and oxygen consumption rate (OCR) were measured using the Seahorse XFe96 analyzer (Agilent Technologies). The unbuffered XF assay medium was produced by adding 0.2 mM sodium pyruvate, 11 mM glucose, and 2 mM GlutaMAX to XF Base Medium and adjusting the pH to 7.4 ± 0.1. The concentrations of TAK-994 were as follows: 3, 10, 30, and 100 μM (final concentration of 0.3 % DMSO v/v). The basal OCR was stabilized for 1 hour, and the test compound was added and incubated for another hour. Subsequently, the mitochondrial stress test was performed by first adding and mixing 2 μM oligomycin, 1 μM FCCP, and 4 μM rotenone/4 μM antimycin A into the prepared assay medium in that order, followed by incubation for approximately 1 hour. Statistically abnormal O_2_ values below 146 mmHg and above 157 mmHg in blank wells were excluded from the analysis, and the frequency of the occurrence was approximately 10 % (3–4 wells out of 36 wells). Non-mitochondrial OCR, basal OCR, proton leak, ATP production, mitochondrial reserve, and maximal oxygen consumption were calculated for each area under the curve (AUC) (Eakins et al., 2016) using the usual definitions.

1. *Genetic data generation and analysis*

DNA from 55 participants from the TAK-994-1501 and the TAK-994-1504 extension studies who consented to DNA analysis were analyzed for genetic associations with DILI-related adverse events. The 55 participants included 5 of the 7 subjects who discontinued due to increases in liver transaminases. DNA was analyzed using the PharmacoScan™ genotype array (Thermo Fisher) and whole-genome sequencing (WGS) to 30X coverage by LabCorp, who provided drug-metabolizing enzyme star allele designations from the PharmacoScan array, and whole-genome single nucleotide variant and short insertions/deletions (InDels) calls from the WGS data. We analyzed the metabolizer status for 3 enzymes potentially involved in TAK-994 metabolism (CYP2C19, CYP2C9, and CYP3A4) and found that all participants with DILI carried genotypes associated with either intermediate or normal metabolic rates, suggesting that the activity of these enzymes was not associated with DILI events. Previously identified DILI-associated single nucleotide polymorphisms (SNPs) in human leukocyte antigen (HLA) and non-HLA regions (Daly et al. 2009; Kowalec et al. 2018; Lucena et al. 2011; Nicoletti et al. 2017; Nicoletti et al. 2019; Parham et al. 2016; Singer et al. 2010; Suvichapanich et al. 2019; Urban et al. 2012) were interrogated using the WGS data. We examined whether those 15 previously identified DILI-associated SNPs were associated withTAK-994-induced DILI using logistic regression among either White participants or all participants. We did not observe a trend of association (p<0.05) for any of the SNPs.

**Supplementary Tables and Figures**

**Supplementary Table 1.** *In vitro* assay detection of clinical DILI.

|  | **TAK-475**  **IC_50_ (μM)** | **TAK-875**  **IC_50_ (μM)** |
| --- | --- | --- |
| HepG2 cytotoxicity for 72 hours | >100 | >100 |
| HepG2 Glu/Gal cytotoxicity for 24 hours | >100 | >100 |
| Liver microtissue toxicity for 14 days | 58.6 | 12.7 |

DILI, drug-induced liver injury; Gal, galactose; Glu, glucose.

**Supplementary Table 2.** Oral gavage toxicity studies of TAK-994 in rodents and non-human primates.

|  | **Dose**  **(mg/kg)** | **ALT**  **(IU/L)** | **AST**  **(IU/L)** | **GLDH (IU/L)** | **Histopathological Liver Findings** |
| --- | --- | --- | --- | --- | --- |
| **Rat 26-week, mean (SD)^a^** | | | | | |
| **Male** | 0 | 32 (11) | 60 (18) | 22 (25) | No adverse findings |
|  | 50 | 29 (6) | 53 (8) | 12 (8) | No adverse findings |
|  | 150 | 38 (31) | 53 (11) | 12 (7) | No adverse findings |
|  | 1000 | 31 (7) | 50 (11) | 7 (4) | No adverse findings |
| **Female** | 0 | 32 (7) | 61 (13) | 23 (27) | No adverse findings |
|  | 50 | 35 (12) | 64 (38) | 41 (78) | No adverse findings |
|  | 150 | 37 (8) | 53 (13) | 12 (14) | No adverse findings |
|  | 1000 | 44 (14) | 51 (6) | 6 (4) | No adverse findings |
| **Monkey 39-week, mean (SD)^b^** | | | | | |
| **Male** | 0 | 31 (13) | 29 (13) | 14 (4) | No adverse findings |
|  | 60 | 42 (20) | 30 (12) | 12 (3) | No adverse findings |
|  | 200 | 32 (2) | 23 (7) | 13 (1) | No adverse findings |
|  | 1000 | 33 (9) | 22 (4) | 13 (3) | No adverse findings |
| **Female** | 0 | 42 (18) | 26 (5) | 14 (6) | No adverse findings |
|  | 60 | 35 (9) | 31 (6) | 11 (2) | No adverse findings |
|  | 200 | 28 (9) | 27 (12) | 11 (1) | No adverse findings |
|  | 1000 | 34 (10) | 24 (5) | 12 (1) | No adverse findings |
| **NonTg rasH2 mice 4-week, mean (SD)^c^** | | | | | |
| **Male** | 0 | 22 (3) | 37 (3) | 8 (1) | No adverse findings |
|  | 300 | 99 (57)* | 79 (34)* | 104 (68)* | Single cell necrosis of hepatocytes |
|  | 2000 | 337 (409)* | 211 (299)* | 331 (356)* | Single cell necrosis of hepatocytes |
|  | 4000 | 1164 (330)* | 443 (150)* | 1303 (408)* | Single cell necrosis of hepatocytes |
| **Female** | 0 | 25 (6) | 51 (9) | 9 (2) | No adverse findings |
|  | 300 | 175 (79)* | 163 (65)* | 172 (66)* | Single cell necrosis of hepatocytes |
|  | 2000 | 581 (278)* | 317 (203)* | 562 (324)* | Single cell necrosis of hepatocytes |
|  | 4000 | 1242 (604)* | 473 (216)* | 1384 (585)* | Single cell necrosis of hepatocytes |

^a^All dose levels had 15 animals per sex. No statistical difference was observed between the TAK-994 and control groups.

^b^All dose levels had 4 animals per sex. No statistical difference was observed between the TAK-994 and control groups.

^c^All dose levels had 10 animals per sex.

*p<0.05 using Shirley-Williams test.

**
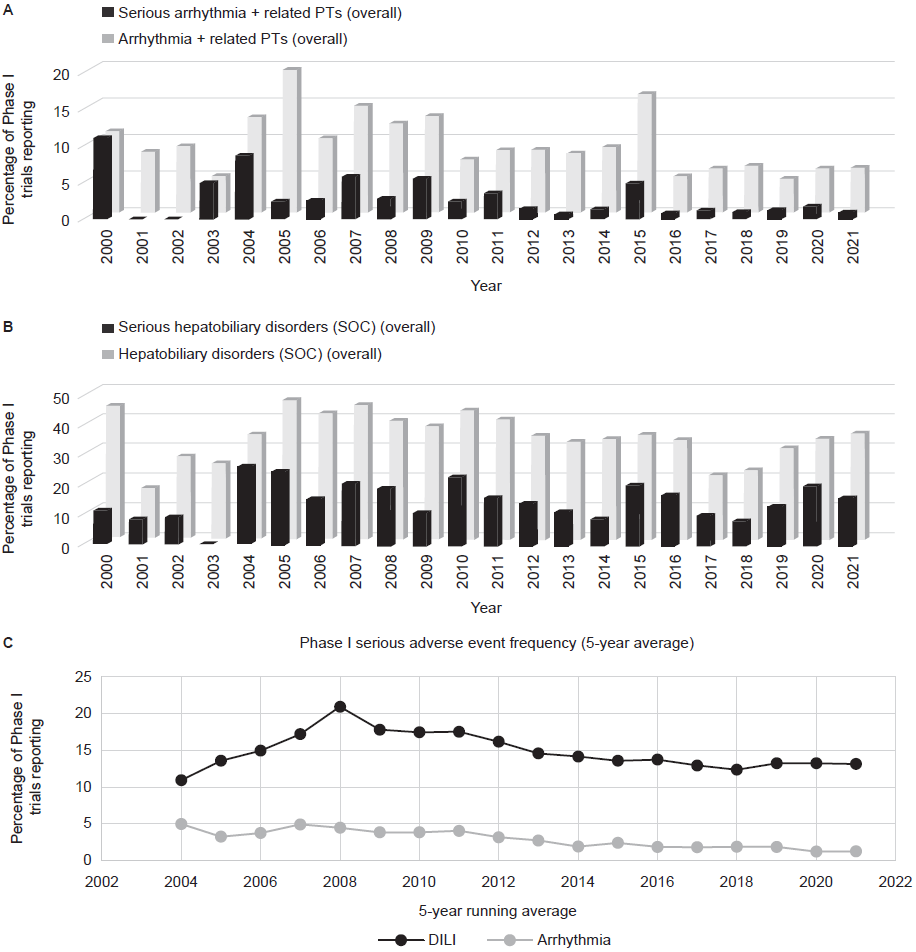
**

**Supplementary Figure 1.** Frequency of arrhythmia and hepatotoxicity in phase I clinical trials. The frequency of (A) arrhythmia and serious arrhythmia (including serious and grade 3 or higher reported events) and (B) hepatobiliary and serious hepatobiliary adverse events remained relatively unchanged over time from 2000–2021 in small-molecule phase I clinical trials. Estimated number of phase I trials per year where the studied adverse events were reported, obtained from the analysis of the safety data of 1,695 phase I clinical trials from published articles and clinical trial registries (limited to 1 study per molecule). (C) The 5-year average frequency of hepatotoxicity serious adverse events remained steady from 2000–2021 in small-molecule phase I clinical trials reporting serious hepatobiliary adverse events. Figure based on data obtained from OFF-X (Clarivate 2022; Monticello et al. 2017; Olson et al. 1998). PTs, preferred terms; SOC, system organ class.

**
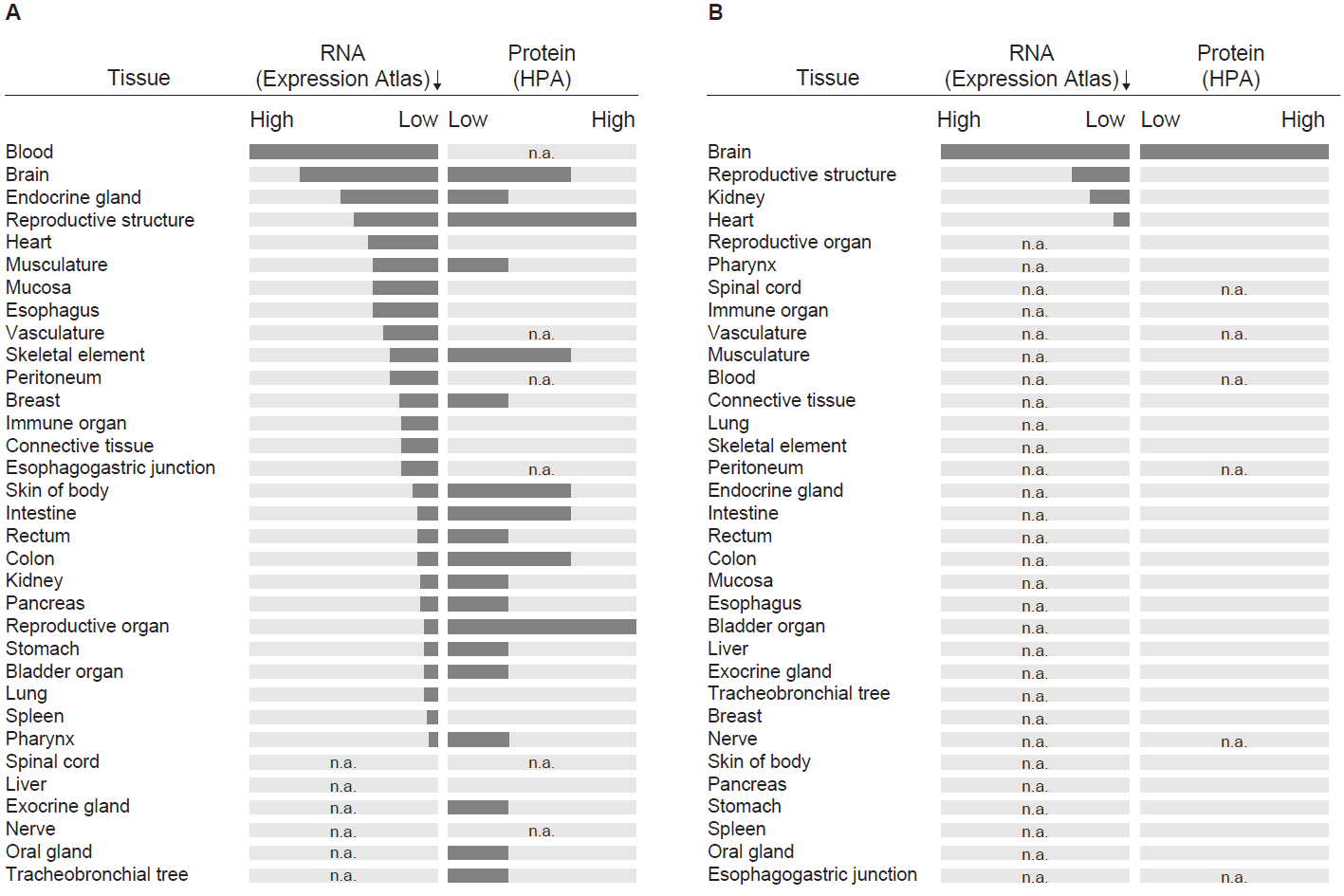
**

**Supplementary Figure 2. OX2R RNA/protein expression in human liver are low/undetectable.** RNA and protein expression of (A) HCRTR1/OX1R and (B) HCRTR2/OX2R in various human tissues. Expression from ProteinAtlas (The Human Protein Atlas: HCRTR2), as of January 26, 2022. HCRTR[1/2], hypocretin receptor 1/2; OX[1/2]R, orexin receptor 1/2; RNA, ribonucleic acid.

**
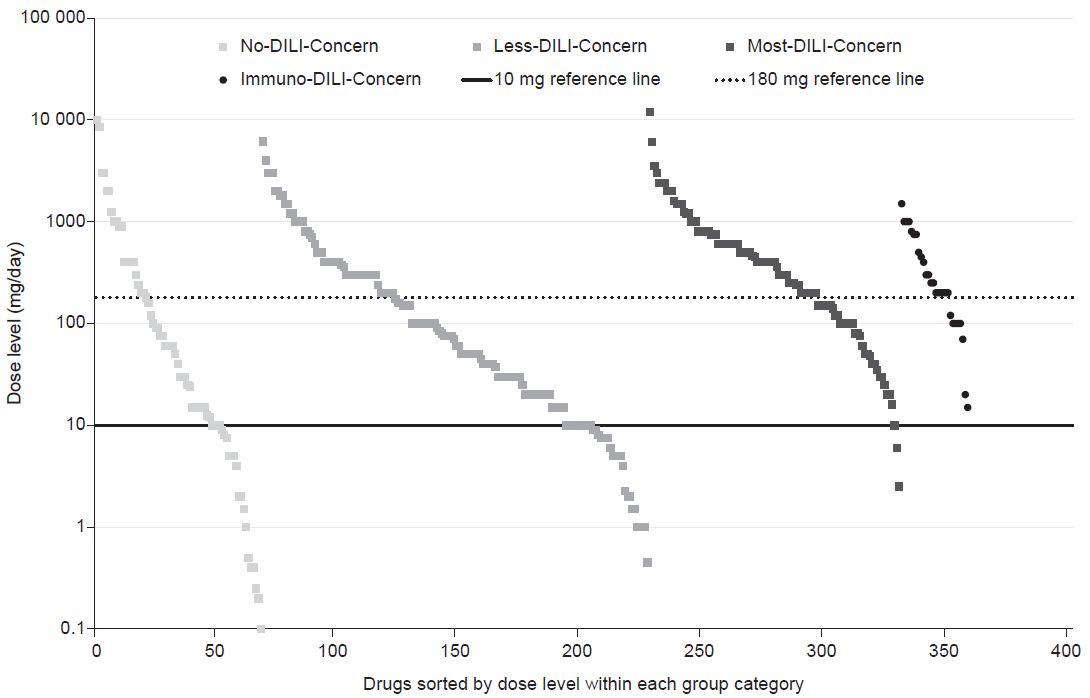
**

**Supplementary Figure 3. Immune DILI has not been observed with small molecules below 10 mg daily dose.** Dose levels for 68 non-DILI concern, 158 less-DILI-concern, 103 most-DILI-concern, and 29 Immuno-DILI-concern drugs were obtained from United States Food & Drug Administration, United Kingdom National Health Service, Medscape, National Institutes of Health LiverTox, and PharmaPendium (Elsevier). DILI, drug-induced liver injury.

**References**

Daly AK, Donaldson PT, Bhatnagar P, Shen Y, Pe'er I, Floratos A, Daly MJ, Goldstein DB, John S, Nelson MR et al. 2009. *HLA-B*5701* genotype is a major determinant of drug-induced liver injury due to flucloxacillin. Nat Genet. 41(7):816-819.

Elsevier. PharmaPendium. [accessed 2024 February 9]. <https://www.elsevier.com/products/pharmapendium>.

The Human Protein Atlas: HCRTR2. Protein Atlas Project; [accessed 2022 January 26]. <https://www.proteinatlas.org/ENSG00000137252-HCRTR2/tissue+cell+type>.

Kohara H, Bajaj P, Yamanaka K, Miyawaki A, Harada K, Miyamoto K, Matsui T, Okai Y, Wagoner M, Shinozawa T. 2020. High-throughput screening to evaluate inhibition of bile acid transporters using human hepatocytes isolated from chimeric mice. Toxicol Sci. 173(2):347-361.

Kowalec K, Wright GEB, Drögemöller BI, Aminkeng F, Bhavsar AP, Kingwell E, Yoshida EM, Traboulsee A, Marrie RA, Kremenchutzky M et al. 2018. Common variation near IRF6 is associated with IFN-β-induced liver injury in multiple sclerosis. Nat Genet. 50(8):1081-1085.

Lucena MI, Molokhia M, Shen Y, Urban TJ, Aithal GP, Andrade RJ, Day CP, Ruiz-Cabello F, Donaldson PT, Stephens C et al. 2011. Susceptibility to amoxicillin-clavulanate-induced liver injury is influenced by multiple HLA class I and II alleles. Gastroenterology. 141(1):338-347.

Nicoletti P, Aithal GP, Bjornsson ES, Andrade RJ, Sawle A, Arrese M, Barnhart HX, Bondon-Guitton E, Hayashi PH, Bessone F et al. 2017. Association of liver injury from specific drugs, or groups of drugs, with polymorphisms in HLA and other genes in a genome-wide association study. Gastroenterology. 152(5):1078-1089.

Nicoletti P, Aithal GP, Chamberlain TC, Coulthard S, Alshabeeb M, Grove JI, Andrade RJ, Bjornsson E, Dillon JF, Hallberg P et al. 2019. Drug-induced liver injury due to flucloxacillin: relevance of multiple human leukocyte antigen alleles. Clin Pharmacol Ther. 106(1):245-253.

Parham LR, Briley LP, Li L, Shen J, Newcombe PJ, King KS, Slater AJ, Dilthey A, Iqbal Z, McVean G et al. 2016. Comprehensive genome-wide evaluation of lapatinib-induced liver injury yields a single genetic signal centered on known risk allele HLA-DRB1*07:01. Pharmacogenomics J. 16(2):180-185.

Rana P, Aleo MD, Gosink M, Will Y. 2019. Evaluation of in vitro mitochondrial toxicity assays and physicochemical properties for prediction of organ toxicity using 228 pharmaceutical drugs. Chem Res Toxicol. 32(1):156-167.

Singer JB, Lewitzky S, Leroy E, Yang F, Zhao X, Klickstein L, Wright TM, Meyer J, Paulding CA. 2010. A genome-wide study identifies HLA alleles associated with lumiracoxib-related liver injury. Nat Genet. 42(8):711-714.

Suvichapanich S, Wattanapokayakit S, Mushiroda T, Yanai H, Chuchottawon C, Kantima T, Nedsuwan S, Suwankesawong W, Sonsupap C, Pannarunothai R et al. 2019. Genomewide association study confirming the association of NAT2 with susceptibility to antituberculosis drug-induced liver injury in Thai patients. Antimicrob Agents Chemother. 63(8).

Thompson RA, Isin EM, Li Y, Weidolf L, Page K, Wilson I, Swallow S, Middleton B, Stahl S, Foster AJ et al. 2012. In vitro approach to assess the potential for risk of idiosyncratic adverse reactions caused by candidate drugs. Chem Res Toxicol. 25(8):1616-1632.

Urban TJ, Goldstein DB, Watkins PB. 2012. Genetic basis of susceptibility to drug-induced liver injury: what have we learned and where do we go from here? Pharmacogenomics. 13(7):735-738.
